# Supplementary material for: A qualitative investigation of views on practice in early support hubs from staff working in care and support roles
Source: PLOS Ment Health. 2026 Jul 31;3(7):e0000657. doi: 10.1371/journal.pmen.0000657 (PMC13426995; doi:10.1371/journal.pmen.0000657)
Supplement: S1 Text — (PDF) [file pmen.0000657.s001.pdf]

Interview Topic Guide

PART 1: CURRENT PROFESSIONAL ROLE AND EXPERIENCE

1. Could you please describe your current role in the early support hub and years of experience working at the early support hub and more broadly in care and support for young people?

   Potential prompts to include:

   - Professional qualifications in health and social care

PART 2: DESCRIBING EARLY SUPPORT HUBS

2. How would you describe what an early support hub and its purpose is to someone who doesn't know about them?

   Potential prompts to include:

   - Who is and isn't the service intended for?
   - What is the purpose of the hub/the intended model?
   - What are the important characteristics of an early support hub?
   - What types of care and support does the service provide for young people?

3. What are the needs of the young people who access the early support hub?

4. For those who have worked in any other mental health services for young people, how are early support hubs different to these?

PART 3: EXPERIENCE OF WORKING IN EARLY SUPPORT HUBS

5. How successful do you think the early support hub is in working with young people with mental health difficulties? What helps or hinders this?

Potential prompts to include:

- What works well for the young people? Which young people benefit?
- What doesn't work well for the young people? Which young people don't benefit?
- What works well for you and your role?
- What doesn't work well for you and your role?
- What impact does the early support hub have?

6. [If appropriate] In what ways is the early support hub better or worse than other mental health services you have worked in?

7. Have you received any training whilst working at the early support hub?

Potential prompts to include: • What training was helpful

or not helpful/relevant?

- Is there any other support that you would like to receive to improve the care and support you provide for young people?

PART 4: FUTURE RECOMMENDATIONS FOR EARLY SUPPORT HUBS

8. Do you feel like you have an active role in service improvement?

Potential prompts to include:

- Improvements regarding service delivery
- Improvements regarding accessibility
- Improvements regarding types of support

9. What improvements would you make to the early support hub, assuming resources are available?

Potential prompts to include:

- What could be improved to make access to the early support hub easier for young people?
- Do the young people using hubs have any needs, which the hubs don't help with, could anything be done to improve this, and how?

- What would you like to improve for people who work at the early support hub in care and support roles?
- Is there anything you have seen in other mental health services that would be useful to provide in your hub?

10. In an ideal world, what do you think an excellent service delivering early support for young people in mental distress in the community should look like?

    Potential prompts to include:

    - Type of care and support offered (e.g. mental health care, physical health care, practical support) and any resources provided • Accessibility and opening hours
    - Who would work there?
    - What kind of environment would it be? (e.g. regarding waiting room, the support rooms, the location)
    - What impact would the early support hub have?

<u>PART 5: CONCLUDING QUESTION</u>

11. Is there anything you would like to add that we have not yet spoken about today?
